# Effects of Age and Initial Risk Perception on Balloon Analog Risk Task: The Mediating Role of Processing Speed and Need for Cognitive Closure

**DOI:** 10.3389/fpsyg.2016.00659

**Published:** 2016-05-06

**Authors:** Maciej Koscielniak, Klara Rydzewska, Grzegorz Sedek

**Affiliations:** Interdisciplinary Center for Applied Cognitive Studies, Department of Psychology, SWPS University of Social Sciences and HumanitiesWarsaw, Poland

**Keywords:** aging, risk taking, BART, initial risk perception, processing speed, need for cognitive closure

## Abstract

According to the dual-process theoretical perspective adopted in the presented research, the efficiency of deliberative processes in decision making declines with age, but experiential processes are relatively well-preserved. The age-related differences in deliberative and experiential processes in risky decision-making were examined in this research by applying the Balloon Analog Risk Task (BART). We analyzed the influence of age on risk acceptance and decision-making performance in two age groups of female participants (younger adults, *n* = 81; older adults, *n* = 76), with additional experimental manipulation of initial risk perception. We predicted and confirmed that aging significantly worsens performance on the behavioral BART measures due to age-related decline in deliberative processes. Older participants were found to exhibit significantly higher risk aversion and lower BART performance, and the effect of age was mediated by cognitive (processing speed) and motivational (need for cognitive closure) mechanisms. Moreover, older adults adapt to the initial failure (vs. success) similarly, as younger adults due to preserved efficiency of experiential processes. These results suggest future directions for minimizing negative effects of aging in risky decision-making and indicate compensatory processes, which are preserved during aging.

## Introduction

The common understanding is that young people are prone to risk taking, while older adults are used as examples of caution and risk aversion (Rolison et al., 2012). However, those stereotypes do not find support in numerous research results and correlational data (Dror et al., 1998; Chou et al., 2007; Samanez-Larkin et al., 2011). Many data indicate the opposite tendency: people aged 55 years or more are actively making investment choices that involve large amounts of risk acceptance (Brown, 2002), and they also occupy the highest-ranking managerial positions among S&P 500 companies (Statistic Brain Research Institute, 2015).

Some researchers (Chaubey, 1974; Deakin et al., 2004) hypothesize gradual age-related decline in quality of functioning within risk and uncertainty conditions, which in turn leads to deficits in decision-making efficiency. However, other psychological scientists do not agree with this notion and report evidence that not only supports no differences between the quality of older and younger adults’ choices in risk and uncertainty conditions (Dror et al., 1998), but also claims that these groups are characterized by similar decision strategies when faced with risky choices (Kovalchik et al., 2005; Bruine de Bruin et al., 2014).

Because the issue of age differences in risky choice is a very complex one, an increasing amount of research is focused on specifying decision task contexts and settings that determine whether older adults seek or avoid risk (for recent reviews, see Henninger et al., 2010; Mata et al., 2011; Cavanagh et al., 2012). We applied the dual-process perspective of age effects on decision-making, recently elaborated by Hess (2015), for a contextual theoretical model. Classical dual-process models (e.g., Bargh, 1994; Epstein, 1994; Kahneman, 2003) differentiate the deliberative processes (effortful, analytical, often verbal, and slow) from the experiential processes (automatic, associative, often affective, and fast). This distinction is important because the empirical evidence (for reviews, see Peters et al., 2007; Hess, 2015) shows that in decision-making, deliberative processes are impaired by aging, but experiential processes are relatively well-preserved. In this research, we examined age-related differences between deliberative and experiential processes in risky decision-making by applying the Balloon Analog Risk Task (BART; Lejuez et al., 2002). Meta-analysis by Mata et al. (2011) indicates that risky tasks such as the Iowa Gambling Task (IGT), the Behavioral Investment Allocation Strategy (BIAS), and the BART, which involve making decisions from experience (i.e., risk information is not explicitly presented; participants estimate risk in a number of trials), showed strikingly different age-related differences. More specifically, in the IGT and BIAS, older adults, rather than younger ones, were more risk-seeking. In contrast, in the BART, older adults were more risk-averse than their younger counterparts.

Therefore, the BART might be currently assessed as the “prototype” task in which older adults prefer risk-avoiding strategies. However, the research on age-related differences in the BART are scarce, and we identified only two studies that directly compared differences between older and younger adults. They yielded only partially consistent findings. The research by Henninger et al. (2010) demonstrated clear age-related impairments in the main behavioral measures (i.e., adjusted number of pumps and number of explosions) in the BART. However, in the research by Rolison et al. (2012), age-related differences in behavioral measures were absent, but the cognitive modeling showed some subtle differences (older adults were more cautious) in the initial perceptions of risk. Obviously, more research is needed to examine the mechanisms of age-related differences in the BART. The first goal of our research was to understand how age-related decline in cognitive deliberative skill, such as processing speed, affects BART performance. The second goal was to examine whether older adults show a relative preservation of experiential processes (affective resilience, Peters et al., 2007; Hess, 2015) and are as adaptive as younger adults in adjusting to a series of incidental failures in the BART. In the next sections, the BART will be succinctly presented; we will then review the existing evidence of age-related differences in deliberative and experiential processes to predict their roles in the relationship between age and BART performance.

Using the computerized BART, participants are to pump a series of virtual balloons, and with each pump, points are gained. Participants decide whether to continue or stop pumping and indicate this by clicking on a button on the monitor. If pumping is terminated, the points gained in that trial will be securely stored in a “bank.” Pumping the balloon too many times carries the risk of the balloon exploding, and the points gained in that trial are lost. However, if the balloon is not pumped enough, the amount of points earned is small. Therefore, risk taking is rewarded only to a certain point, and then it is penalized. This is a reflection of numerous real-life situations.

The BART has been proven to have strong ecological validity because it is a strong predictor of real-life risk taking behavior in a number of populations (Lejuez et al., 2003; Aklin et al., 2005; Hopko et al., 2006; Bornovalova et al., 2009), including older adults (Henninger et al., 2010; Cavanagh et al., 2012; Rolison et al., 2012; Seaman et al., 2015). Lejuez et al. (2002) claimed that the BART is sometimes a more reliable measure of risk-taking behavior than self-reports and demographic data. According to a meta-analysis involving the BART (Lauriola et al., 2014), most publications demonstrated significant correlations between the average adjusted number of pumps and risky behaviors in real life, such as smoking, alcohol and drug use, aggression, participation in unprotected sex, and gambling.

Age-related limitations in deliberative cognitive processes might be plausible mechanisms of impaired BART performance among older adults. When confronted with complex risky decision tasks such as the BART, effective functioning of participants demands active deliberation, such as counting and averaging the number of pumps across trials, suppressing negative feelings caused by balloon explosions, and weighing the pros and cons of further pumping. Hence, there are high demands on cognitive and attentional resources when it comes to effective BART performance. The scientific literature (Engle et al., 2005; Thornton and Dumke, 2005; Salthouse, 2006, 2012; Sedek et al., 2013), indicates a clear and marked monotonic decrease in basic fluid cognitive abilities (such as processing speed, working memory capacity, and fluid intelligence) from early adulthood through middle age to old age, and this is responsible for decreased performance in various cognitive tasks. The processing speed theory developed by Salthouse (1996) and using the Digit Symbol Substitution Test (DSST) as a principal measure, accounts for age-related changes, such as impairment in logical reasoning (Salthouse, 2001; Sedek and von Hecker, 2004), in many cognitive tasks. Using the BART, Henninger et al. (2010) found that older adults have greater aversion to risk than younger adults, thus the quality of their decision-making in that task was significantly lower. In addition, they showed that age-related differences in BART performance are mediated by processing speed. However, cognitive speed was measured using the Digit Symbol test, which is similar to the DSST but has some different characteristics. The processing speed theory (with DSST as a principal measure) developed by Salthouse (1996) accounts for age-related differences in many cognitive tasks. According to this theory, slower speed of executing cognitive operations in older age impairs performance in more complex tasks because of two mechanisms. The key defining feature of the first mechanism (the “time limited” mechanism) is that time constraints cause processing of the initial information to be incomplete and defective. This is in contrast to the second mechanism (the “simultaneity” mechanism) in which processing the initial information is complete but decay causes it to become unavailable in the later stages of processing. Both cognitive mechanisms may be responsible for age-related decline in BART performance. Hence, to replicate and extend previous findings about the influence of age on BART performance (Henninger et al., 2010), the DSST was included as a measure of processing speed.

Another way in which older adults appear to manage lower efficiency in deliberative cognitive processes is through selective engagement in cognitive resources. Older adults may conserve resources by simplifying their interactions with the environment and by limiting both the quantity and complexity of the information to which they attend. This description is based on the classical framework of selection, optimization, and compensation (SOC; Baltes and Baltes, 1990). According to Hess (2000, 2014, 2015), aging is connected with increasing costs of engagement in effortful cognitive activities. As aging proceeds, more resources are necessary to achieve a particular level of performance in an effortful task. Furthermore, a perception of costs results in lack of intrinsic motivation to commit resources to cognitively demanding tasks, and this in turn is reflected in the selection processes of directing and energizing. We measured cognitive motivation by applying the Need for Cognitive Closure Short Scale (NFCC; Kruglanski, 2004). The need for closure has been defined as a desire for a definite answer to a question or for any firm answer rather than uncertainty, confusion, or ambiguity (Kruglanski and Webster, 1996). Aging usually increases scores on the NFCC; however, age effects have varied size effect and are divergent for the different subscales (Blanchard-Fields et al., 2001; Cornelis et al., 2009; Kossowska et al., 2012b). The most consistent age effects are observed for two NFCC subscales: Desire for Predictability and Preference for Order and Structure (Blanchard-Fields et al., 2001; Czarnek et al., 2015). Most previous research has demonstrated that the NFCC is related to a simplified cognitive process involved in decision-making, a limited information search, and a preference for clear and unambiguous judgment (see the recent review in Roets et al., 2015). Several authors (Cornelis et al., 2009; Kossowska et al., 2012b; Roets et al., 2015) suggested the interplay between age-related decline in deliberative processes and age-related increase in the NFCC. Namely, the complexity of encountered tasks in real-world situations often exceeds the cognitive resources of older adults, and this corresponds to the age-related increase in some of the NFCC subscales measuring epistemic motivation (i.e., they are motivated to strive for simplification, more predictability, and order). Our recent study (Czarnek et al., 2015) showed some support for the double mediation of age effect on stereotypic judgments by cognitive and motivational processes. Namely, we found that, compared to younger participants, older adults were more likely to rely on stereotypic inferences when they read a story about out-group members. In addition, the findings showed that making more stereotypical inferences by older vs. younger adults in relation to out-group members was mediated by cognitive (Trail Making Test) and motivational (NFCC) mechanisms. The important research question is whether the influence of aging on some measures of the BART is also independently determined by a decline in mental speed and an increase in the NFCC.

Recent findings (e.g., Queen and Hess, 2010; Hess et al., 2012) and reviews (Peters et al., 2007; Hess and Ennis, 2014; Hess, 2015) showed that age-related differences in decision making tasks are eliminated when decision making performance strongly relies on experiential (e.g., affective) processes. Interestingly, a substantial number of studies on the IGT (Bechara et al., 1994), reflecting the activity of affective cortical structures, did not demonstrate age-related differences (e.g., MacPherson et al., 2002; Kovalchik et al., 2005; Wood et al., 2005; but see clear age-related differences in Denburg et al., 2009). The most inspiring for us were the detailed analyses of age-related differences found by Wood et al. (2005). They found that younger adults applied both deliberative and experiential processes in forming their choices between more- and less-risky card decks in the IGT. However, older adults’ choice behaviors were guided more by recent losses and gains, suggesting greater employment of experiential processes. This strategy was quite effective in this study because both older and younger adults performed similarly in the IGT.

Inspired by these analyses of the IGT by Wood et al. (2005), we decided to experimentally manipulate experienced failures and successes in preliminary trials of the BART. Specifically, we procedurally increased the participant’s initial failures (the first three balloons in the task explode almost immediately, and participants hardly earn any points) or initial successes (the first three balloons in the task explode after more than 20 pumps, hence participants earned a lot of points). As seen by Wood and associates, such manipulation should be guided by experiential processes and affect in both younger and older participants similarly. According to recent research applying advanced cognitive modeling to explain age-related differences in BART performance (Cavanagh et al., 2012; Rolison et al., 2012), such manipulation should initially significantly differentiate adopted decision strategies (more cautious after initial failures, more risky after initial successes), but this effect should be of short duration (with increased experience).

The impact of experiential processes in BART performance is related to the potential role of positive emotions in explaining age-related differences in decision-making. Improved affective experience has been found to be associated with aging (Carstensen, 2006; Charles and Carstensen, 2010; Sedek et al., 2014). Moreover, studies show that affect influences search tendencies and therefore, decision-making. As an important example, increased positive mood among older adults leads to less information search in sequential choice tasks (von Helversen and Mata, 2012), thereby worsening performance. Because the BART is undoubtedly a sequential decision-making task, we also examined whether positive affect influences BART performance.

In the BART, women tend to pump the balloon substantially less than men (Lejuez et al., 2002). To eliminate complex interactions that may be evoked by the unexpected influence of participants’ sex, we decided to conduct this study using a group of only women, those commonly regarded as more risk averse than men.

To succinctly summarize our main predictions: (1) Aging significantly worsens performance on behavioral BART measures because of age-related decline in deliberative processes; (2) Experiencing incidental failures (vs. successes) at the beginning of the task affects BART performance (at least in the first phase of the task). However, older and younger adults should adapt similarly to this manipulation because of preserved efficiency of experiential processes.

## Materials and Methods

### Participants

One-hundred fifty-eight female participants from two specific age groups were invited to participate in the study. The group of younger adults consisted of 81 students aged 18–23 years (*M* = 19.86 years; *SD* = 1.00) recruited from first- and second-year university courses in exchange for partial credit. The group of older adults included 77 participants aged 65–80 years (*M* = 69.19 years; *SD* = 4.32) who volunteered to participate through a recruitment company. Participants who performed best on the BART received material prizes, namely optional books or shopping vouchers of the equal nominal value of $12.

Inclusion criteria were possession of a basic level of computer literacy, allowing for comfortable and free performance of the computerized task and use of corrective lenses (either eyeglasses or contact lenses) when vision defects warranted it so that effects of poor vision were minimized.

Information about education level in terms of the years of formal education completed was participant-provided, and no significant differences were found between the groups. Among the older adults, mean (*SD*) level of education was 13.43 (3.06) years, whereas among the younger adults, it was 13.59 (2.05) years (*F <* 1).

### Cognitive, Motivational, and Affective Measures

Mental speed was measured using the DSST (Wechsler, 1981), a classical measure of processing speed that involves substitution of digits with symbols according to a given template within a time limit.

An additional measure of the ability to engage in effortful inference processes and avoid judgment biases was determined by applying the Cognitive Reflection Test (CRT; Frederick, 2005), a three-item measure (e.g., “a bat and a ball cost $1.10 in total; the bat costs $1.00 more than the ball; how much does the ball cost?”).

The NFCC (Kruglanski and Webster, 1996) was measured using the Polish version (Kossowska et al., 2012a). Participants were asked to rate themselves on 15 items describing various real-life situations using a Likert scale ranging from 1 (*totally disagree*) to 6 (*totally agree*). Additionally, the Polish version (Fajkowska and Marszał-Wiśniewska, 2009) of the Positive and Negative Affect Schedule – Expanded Form (PANAS-X; Watson and Clark, 1994) was applied to measure current affective state. The instrument consisted of 40 affective words (such as lonely, concentrated, and happy) and participants rated how well each item described their current mood on a seven-point scale ranging from 1 (*not at all*) to 7 (*very much*). Positive and negative affect scores were calculated by taking the mean rating of either positive or negative items.

### Balloon Analog Risk Task

**FIGURE 1:**
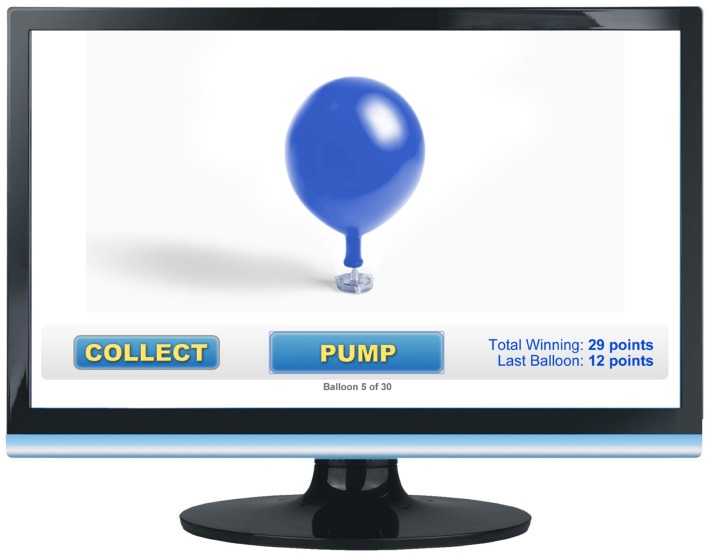
**Balloon Analog Risk Task**.

To measure risky behavior, a version of the BART (Lejuez et al., 2002) programmed in Java Script language (interface shown in **Figure 1**) was used. The task consisted of two series carried out approximately 15 min apart. Each comprised 30 trials, and in each trial, the participant was presented with a realistic picture of a balloon on the computer monitor. The construction of BART was the same as described in detail by Lejuez et al. (2002). The series were most similar to yellow balloon series (Lejuez et al., 2002) in which to maximize earnings participants should learn to pump each balloon approximately 16 times, ignoring the explosions. In our series, the average break point was 15 (30 trials with the explosions between 1 and 30 pumps); therefore, to maximize earnings participants should learn to pump each balloon approximately 15 times. The task was to virtually inflate the balloon by clicking on the button on the monitor marked ‘PUMP’ and it was done in a self-paced manner. Each time this was done, the balloon visually grew in size, and the participant received one virtual point. At any time, the participant could stop inflating the balloon and secure the points in a virtual bank. This was done by clicking on a button marked ‘COLLECT.’ Each pump increased the risk of losing all the points gained in the current trial, but it did not affect points already secured in the bank. The balloon would explode at a random point in time between the first and thirtieth pumps (the maximum number of pumps was 30).

### Experimental Manipulation of the BART

We experimentally modified the BART to influence the participant’s initial failures or successes. The task included two series requiring inflation of 30 balloons in each. The number of pumps until a balloon exploded was randomized (trials with the explosions between 1 and 30 pumps) except for the first three trials, which were used for the experimental manipulation. These three trials were either *unlucky* (explosions with pumps 3, 4, and 1) or *lucky* (explosions with pumps 25, 27, and 28), and participants received both series in a random order during the course of the task. We used the two versions of random orders for the trials (4–30) and these versions were counterbalanced across participants for the series with the lucky and unlucky trials. The effectiveness of this employed manipulation was confirmed using within subjects analysis of variance, where the dependent variable was the number of points earned in the first three trials in two series (unlucky vs. lucky series): in the unlucky series, participants earned *M* = 0.14 points (*SD* = 0.33), while in the lucky series, participants earned *M* = 10.62 points (*SD* = 4.80), *F*(1,157) = *746.48, p* < 0.001, η^2^ = 0.826. The effectiveness of this employed manipulation was additionally confirmed using within subjects analysis of variance, where the dependent variable was the percentage of explosions in the first three trials in two series (unlucky vs. lucky series): in the unlucky series, 95% of the first three balloons exploded, while in the lucky series, 6% of the first three balloons exploded, *F*(1,157) = 3645.43, *p <* 0.001, η^2^ = 0.959.

### Principal BART Measures

The BART provides various types of data that describe risk tolerance and preferred decision-making strategies. We used three primary measures to assess risk-taking behavior: adjusted number of pumps, total points earned, and total number of explosions. These measures were originally suggested by Lejuez et al. (2002).

The most commonly used measure from the BART is the adjusted number of pumps, calculated as the average number of pumps on the balloons that did not explode. For the purpose of statistical analysis, this is the most favorable variable because incorporating all trials (including those in which the balloon exploded) would result in using trials where participants did not make their own decisions, but were forced to stop pumping (Hunt et al., 2005). Thus, using the adjusted number of pumps describes a relatively unbiased risk attitude (Seaman et al., 2015). Making a deliberate decision to stop pumping is an example of a non-punitive form of risk-taking behavior. On the contrary, the total number of explosions represents the frequency of making a decision that exceeded the optimal risk level and which was followed by immediate punishment (explosion and loss of points; Hunt et al., 2005).

The total number of points collected during both series (excluding trials introduced for experimental manipulation) was the final measure determined using the BART. This dependent variable is often treated as an effectiveness index of decision-making strategy because maximization of earnings requires identifying the optimal level of risk. However, it is not a perfect measure of risk propensity because identical earnings may be gained both when a participant earns little for each balloon, but causes few explosions and when she earns large but infrequent reward, resulting in many explosions.

Despite the fact that each of these measures describes a different aspect of decision-making under risk and uncertainty, there is evidence they independently (in most experimental procedures) produce similar findings (Lejuez et al., 2003).

### Procedure

All participants gave informed consent for participation in study procedures. Tests were completed in a room with 7–9 other participants from the same age group, who were simultaneously doing these tests in the same order. Each participant was provided a desk so that paper-and-pencil measures could be completed as well as a computer equipped with the Windows 7^®^ operating system and a 22-inch monitor. Monitors were set in a way that prevented participants from seeing other monitors and from communicating with one another. Three experimenters were present at all times during study procedures, ensuring close and individual monitoring of study participants.

The first series of the BART was completed using the computer. Following this, the DSST, measuring mental speed of processing information, was completed. Participants were then instructed to complete the PANAS-X to self-report current affective state. The NFCC and CRT were then completed. Finally, the second series of the BART was completed. After all participants finished the course of activities, those who scored the most points on the BART in each group received a prize, which was made explicit to participants prior to completion of study procedures. The entire study took 30–50 min to complete.

## Results

The effects of Age on processing speed, Cognitive Reflection Test, Need for Cognitive Closure, and PANAS X Scale are summarized in **Table 1.** Preliminary analyses showed that the Order of Series (unlucky first, lucky first; between subjects variable) did not yield any significant results as concern main effect and interaction effects with any other variables for all dependent measures, hence we omitted this variable in factorial design.

**Table 1:** Participant characteristics.

Measures	Younger adults (*N* = 81F)		Older adults (*N* = 77F)		Statistical test		
			
	*M*	*SD*	*M*	*SD*	*F*	*p*	$\eta_{p}^{2}$
DSST^a^	120.11	13.77	74.34	21.44	234.37	<0.001	0.616
CRT	1.00	1.04	0.58	0.70	8.67	0.004	0.053
NFCC_2	21.77	4.83	28.12	5.39	61.00	<0.001	0.281
PANAS-X Scale							
General positive affect	31.89	6.40	35.88	6.55	15.02	<0.001	0.088
General negative affect	15.15	6.19	13.75	5.93	2.09	0.150	0.013


*DSST, Digit Symbol Substitution Test; CRT, Cognitive Reflection Test; NFCC_2, sum of the two subscales of Need for Cognitive Closure (Desire for Predictability and Preference for Order and Structure). ^a^Sample size for DSST was 71 for younger and 77 for older adults because data for this measure was not collected from 10 younger adults because of experimenters’ omissions.*

### Risk Taking

As seen in previous studies, the principal measure of risk-taking behavior using the BART was the adjusted number of pumps; two additional measures were total points earned and number of explosions.

A 2 × 2: (Age [younger adults, older adults] × First Trials [unlucky, lucky; within subject variable]) mixed analysis of variance (ANOVA) of these dependent measures yielded only the main effects of Age (see **Table 2**) and the systematic main effect of First Trials (see **Table 3**).

**Table 2:** Analysis of variance main effects of the Age variable on risk indicators in BART task (2 × 2 schema: Age × First Trials).

Measures	Younger adults		Older adults		Statistical test		
			
	*M*	*SE*	*M*	*SE*	*F*	*p*	$\eta_{p}^{2}$
Adjusted number of pumps	8.77	0.23	7.18	0.24	23.07	<0.001	0.129
Total number of points	157.03	2.94	143.62	3.01	10.16	0.002	0.061
Total number of explosions experienced	9.35	0.41	7.23	0.42	12.88	<0.001	0.076


**Table 3:** Analysis of variance main effects of First Trial variable on risk indicators in BART task (2 × 2 schema: Age × First Trials).

Measures	Failure series		Success series		Statistical test		
			
	*M*	*SE*	*M*	*SE*	*F*	*p*	$\eta_{p}^{2}$
Adjusted number of pumps	7.25	0.18	8.70	0.19	76.31	<0.001	0.328
Total number of points	144.76	2.73	155.88	2.73	10.26	0.002	0.062
Total number of explosions experienced	7.14	0.30	9.45	0.34	76.25	<0.001	0.328


Results for the main effects of the Age variable clearly indicate that older female adults adopt higher risk avoidance behavior than younger female adults, performing worse on the BART (average adjusted number of pumps was lower, fewer points earned, lower number of explosions). The main effects of First Trials consistently indicate that both older and younger female adults adopted much riskier behavior in BART performance when the first trials in the series were lucky (consequently average adjusted number of pumps was higher, more points earned, higher number of explosions) rather than when they were unlucky.

### Adaptations to Unlucky and Lucky Series

To determine in more detail the influence of introduced experimental manipulation, we carried out a mixed model ANOVA to compare the behaviors of participants immediately after manipulation (at the beginning of the series), in the middle, and at the end of the series. To accomplish this, an additional within subject variable (Phase of Series) was created, and the average adjusted number of pumps in in three subsequent phases of nine trials following experimental manipulation (first phase: trials 4–12; middle phase: trials 13–21; final phase: trials 22–30) was determined.

A 2 × 2 × 3: (Age [younger adults, older adults] × First Trials [unlucky, lucky; within subject variable] × Phase of Series [first, middle, final; within subject variable]) mixed ANOVA of the adjusted number of pumps in nine trials yielded two main effects and one interaction effect.

A main effect of Age was found, *F*(1,154) = 16.60, *p* < 0.001, $\eta_{p}^{2}$ = 0.097, showing that adjusted number of pumps was lower in older adults (*M* = 7.87) than in younger adults (*M* = 9.64). A main effect of First Trials was also found, *F*(1,154) = 100.82, *p* < 0.001, $\eta_{p}^{2}$ = 0.396, showing that adjusted number of pumps was lower when the unlucky series was presented (*M* = 7.76) than when the lucky series was presented (*M* = 9.76).

**FIGURE 2:**
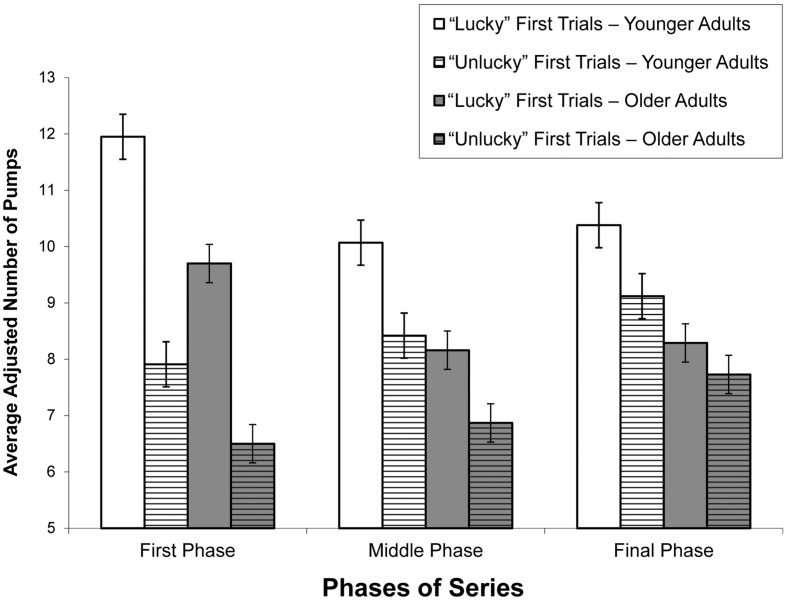
**Adjusted average number of pumps as a function of First Trials, Phase of Series, and Age.** Error bars represent standard errors.

The main effect of First Trials was qualified as significant interaction effect, namely First Trials × Phase of Series, *F*(1,154) = 42.95, *p* < 0.001, $\eta_{p}^{2}$ = 0.218 (see **Figure 2** – for completeness, the age effect is included). As shown in **Figure 2**, the adjusted number of pumps during the initially unlucky series was lowest in the first phase and then gradually increased (*p* < 0.05) in the subsequent phases. However, the adjusted number of pumps in the initially lucky series was higher in the first phase than in the other phases (*p* < 0.001) and similar in the middle and final phases. At the beginning of a series, the adjusted number of pumps was higher if the series was initially lucky than if it was initially unlucky ($\eta_{p}^{2}$ = 0.486). Those differences were gradually decreasing ($\eta_{p}^{2}$ = 0.149 in the middle phase; $\eta_{p}^{2}$ = 0.114 in the final phase). However, all the differences in the adjusted number of pumps between lucky and unlucky series across all phases were highly significant (*p* < 0.001).

Very similar results were found when we analyzed total number of explosions experienced (and points earned) in the first, middle, and final phases; therefore, we do not report these results to avoid redundancy of information.

The age of the study participants does not statistically significantly interact with any of the other variables. Therefore, it can be concluded that aging does not result in significant impairment on BART performance after incidental failure or incidental success experience.

### Mediating Mechanisms

Preliminary correlational analyses showed that only processing speed (measured by DSST) and need for cognitive closure (NFCC) were significantly correlated with BART measures (**Table 4**); hence only those variables were considered potential mediators in the relationship between age and BART performance.

**Table 4:** Correlation table of dependent, independent, and demographic variables.

Variable	1	2	3	4	5	6	7	8
(1) adjPump	–							
(2) Earnings	0.639ˆ***	–						
(3) Explosions	0.913ˆ***	0.355ˆ***	–					
(4) Age group	-0.280ˆ***	-0.247ˆ**	-0.276ˆ***	–				
(5) DSST	0.361ˆ***	0.331ˆ***	0.313ˆ***	-0.785ˆ***	–			
(6) CRT	0.086	0.058	0.072	-0.229ˆ**	0.210ˆ*	–		
(7) NFCC_2	-0.235ˆ**	-0.044	-0.288ˆ***	0.530ˆ***	-0.369ˆ***	-0.221ˆ***	–	
(8) Positive affect	-0.097	-0.035	-0.137	0.296ˆ***	-0.231ˆ**	-0.078	0.177ˆ*	–


*adjPump = Adjusted balloon pumps, average number of pumps’ Earnings = total points collected’ Explosions = number of balloon explosions experienced; Age Group = Older Adults vs. Younger Adults; DSST = Digit Symbol Substitution Test; CRT = Cognitive Reflection Test; NFCC_2 = sum of the two subscales of NFCC (Desire for Predictability, and Preference for Order and Structure); Positive and Negative Affect = PANAS-X scores. ^∗^*p* < 0.05, ^∗∗^*p* < 0.01, ^∗∗∗^*p* < 0.001.*

Results of the positive affect scale are statistically significantly differentiated by age (**Table 1**), confirming the existence of a positivity effect (Carstensen et al., 1999). However, positive affect did not correlate with any measures determined by the BART (**Table 4**). Similarly, the CRT (Frederick, 2005), assumed to measure engagement in effortful inference processes, had higher scores in the younger group (**Table 1**); however they, too, were not correlated with BART measures (**Table 4**).

To verify the hypothesis that there is no direct relationship between participant age and their behavior under risk and uncertainty (after controlling for mechanisms explaining such a relationship), a mediation analysis using Process software (Hayes, 2013) was conducted. It is important to underline that such mediation analyses are not seen at any rate as causal developmental change but only as the examination of direct and indirect influence of age differences on risky decision choice. As described by Hayes and Scharkow (2013) for optimal tests of the indirect effect in statistical mediation analysis, we applied the recommended percentile bootstrap CI method. The DSST score was the measure of processing speed of cognitive functioning, the sum of the two NFCC subscales was the measure of motivation to select incoming information.

**FIGURE 3:**
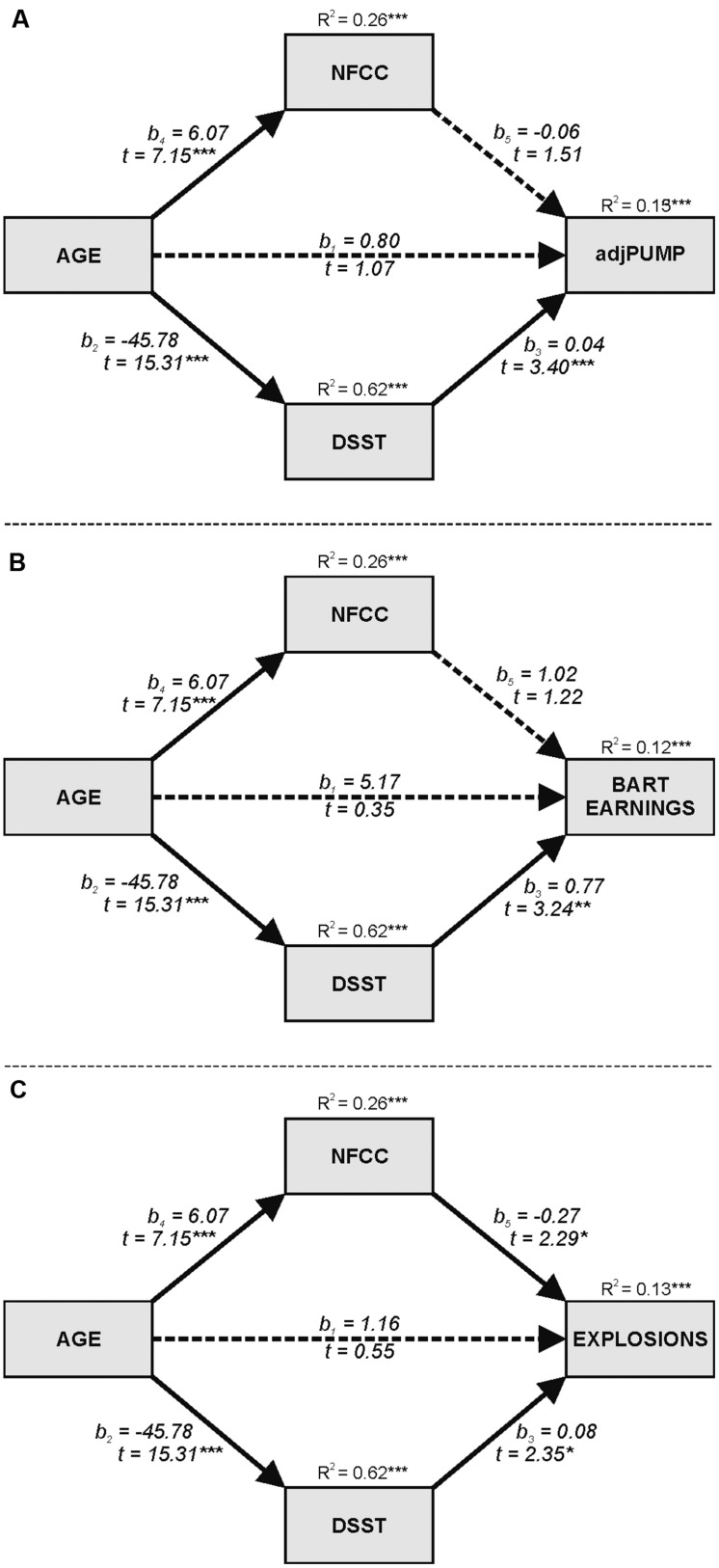
**Models depicting mediated effects of age differences via processing speed (DSST) and Need for Cognitive Closure (NFCC_2) on: **(A)** adjusted number of pumps; **(B)** total points; **(C)** number of explosions in BART experiment.** Bold arrows indicate significant indirect effects; dashed arrows indicate non-significant effects. Entries are unstandardized regression coefficients with *t*-test values. ^∗^*p* < 0.05, ^∗∗^*p* < 0.01, ^∗∗∗^*p* < 0.001.

The strongest mediator of the relationship between age and risk acceptance measures is processing speed (NFCC was reliable mediator only for number of explosions measure). In **Figure 3A**, the mediation influence of these variables on the relationship between age and adjusted number of pumps is shown. Bootstrapping (*n* = 10000 bootstrap resamples, 95% confidence intervals) revealed the significant indirect effect of aging on risk-seeking via DSST (*b*_2_ × *b*_3_ = -1.86; *BootLLCI* = -2,91; *BootULCI* = -0.84), while the direct effect becomes statistically insignificant in this model (*b*_1_ = 0.80; *p* = 0.29).

The mediation mechanism is shaped very similarly when analyzing the influence of age on total score, expressed as earned points (see **Figure 3B**), and yields analogous results: an insignificant direct effect of age on the dependent variable (*b_1_* = 5.17; *p* = 0.73) and a strong mediation path via processing speed (*b_2_* × *b*_3_ = -35.26; *BootLLCI* = -58.41; *BootULCI* = -13.44). These findings clearly demonstrate that risk acceptance and effectiveness of choices made under risk and uncertainty do not depend directly on age, but on the impairment of cognitive functions, which are characteristic to aging.

This model is even more extensive when applied to the relationship between age and the number of explosions experienced in the BART. While a strong main effect of this relationship is shown in **Table 2**, it becomes insignificant (*b*_1_ = 1.16; *p* = 0.58) after introducing two mediating variables: the processing speed of information and the need for closure (see **Figure 3C**). The DSST is again a mediating factor (*b*_2_ × *b*_3_ = -3.59; *BootLLCI* = -6,72; *BootULCI* = -0.41); however, the scale of this mediation is additionally increased by the NFCC (*b*_4_ × *b*_5_ = -1.63; *BootLLCI* = -3,28; *BootULCI* = -0.08). A need for cognitive closure expresses the epistemic motivation, which significantly increases with age, resulting in more judicious pumping of balloons, thus resulting in fewer explosions.

## Discussion

This study used the BART to investigate the influence of aging on the specificity of decision-making under risk and uncertainty among women. The primary result was a strong negative effect of age on all decision-making aspects of the task, affecting both the degree of risk tolerance and efficiency of performance. This agrees with previous research findings on age-related limitations of BART performance (Henninger et al., 2010; Cavanagh et al., 2012; Rolison et al., 2012). More generally, these findings confirmed the notion of Mata et al. (2011) that older adults demonstrate risk aversion in the risky choice context in which risk-seeking would be a more effective strategy.

The fact that incidental failures cause initial increases in precaution similarly among younger and older adults may be because it is a fairly common life experience, especially in the context of consumer behavior. For instance, a batch of light bulbs of a given brand may easily overheat. As a result, consumers may react by automatically using caution when purchasing products of that brand, but they may gradually and slowly change with more positive experiences. It is in line with a number of studies (Queen et al., 2012; Hess et al., 2013), which demonstrate that age differences disappear in the domain of consumer behavior regarding well-known products. It is therefore explained by possessing life experiences and by well-preserved experiential processes among older adults. Similarly, incidental successes are usually followed by temporary increases toward more risky behaviors. For example, in a recently published study conducted in natural settings, it was found that after a basketball team’s victory, residents of the town purchased substantially more lottery tickets (Otto et al., 2016).

### The Mediating Roles of Processing Speed and Need for Cognitive Closure

Further analyses showed that processing speed (as measured by the DSST) mediates age-related differences in the basic measures of the BART: the adjusted number of pumps, the earned points, and the number of explosions. The relationship between age and explosions was also mediated (double-unrelated mediators) by the need for closure measure (NFCC_2). These findings confirmed the results of previous research (Henninger et al., 2010), which used a different measure of processing speed. Additionally, our analyses identified full mediations in terms of Baron and Kenny’s (1986) classical mediation model: the direct and strong effect of age on BART measures disappeared when processing speed (and in case of explosions, also the need for closure) was included in the mediation model. On a more general level, these findings are consistent with the general role of processing speed (Salthouse, 1996, 2001) as the mediator of the relationship between age and performance of complex cognitive tasks. More specifically, these findings agree with the results described in many recent studies (e.g., Finucane et al., 2005; Mata et al., 2007; Frey et al., 2015) – that processing speed and some other cognitive variables (e.g., working memory) are important mediators of age-related differences in complex decision-making tasks.

The construct of the need for cognitive closure, which increases with age, also deserves more applications in the domain of aging and risky decision-making. Researchers emphasize its importance at every stage of life (Roets et al., 2015); however, it appears to be of special importance in older age. Individuals with high NFCC are intolerant of confusion and uncertainty, and therefore are inclined to make rapid decisions. On the other hand, low-NFCC individuals are motivated to analyze situations in a systematic manner, consider alternative options, and make complex decisions (Kruglanski and Webster, 1996). Most previous research has demonstrated that NFCC is related to a simplified cognitive process involved in decision-making, a limited information search, and a preference for clear and unambiguous judgment. More importantly, it has shown that high-NFCC individuals are characterized by fewer cognitive resources that can be allocated to present activities (Kossowska, 2007; Kossowska et al., 2010). Thus, in our recent research (Czarnek et al., 2015), we found that aging decreased processing speed and cognitive flexibility as measured by the Trail Making Test and increased NFCC (the same two NFCC subscales used here) and that both factors mediate the relationship between age and drawing stereotypical inferences about out-groups. In this current study, we again found the double-mediation of a cognitive factor (mental speed) and a motivation factor (need for cognitive closure) of the relationship between age and the number of explosions experienced during the BART. Further research is needed to elucidate the roles of both cognitive and motivational mechanisms in age-related differences in risky choices.

### The Role of Initial Risk Perception in the BART

In-depth analyses provided more information on participants’ behaviors connected with the influence of incidental failure experiences (three unlucky trials) on decision-making under risk in the BART. A consistent pattern of results was acquired, suggesting that study participants pump the balloon far more boldly after a safe beginning than after experiencing incidental failure. However, this influence gradually disappears as experience is gained, and the effect of previously experienced failure becomes weaker. Generally, this pattern of results is partially consistent with the predictions of cognitive modeling studies (Cavanagh et al., 2012; Rolison et al., 2012; Seaman et al., 2015) that initial perceptions of high risk might impair BART performance. The new and intriguing evidence is that such manipulation of incidental failure is very strong and lasts across the entire series. The additional advantage of this study was that the strength of this effect was confirmed by experimental manipulation.

This pattern of results does not differ statistically in both age groups, supporting the existing hypotheses that older adults have the ability to adapt their behavior to changing conditions (Queen et al., 2012). Older women who were subjected to incidental failures at the beginning were not only able to adjust their balloon pumping strategies to further task conditions equally efficiently as younger women, but also stabilized the number of pumps at the very end to levels equaling those of participants subjected to lucky beginnings. Maintaining such flexibility in decision-making is undoubtedly an example of a mechanism compensating for some cognitive limitations.

### Limitations and Future Research

Extended replications and longitudinal paradigms are needed to establish whether the double-mediation models (including both cognitive and motivational variables) are reliable for the BART and whether they could be extended to other risky decision paradigms, such as the IGT, which showed opposite tendencies in aging (cf. Mata et al., 2011). Also, it would be important to determine whether experimental manipulation with incidental failures (or successes) will replicate both very strong effects and gradual adaptations in other risky choice paradigms and to confirm the preserved ability of older adults to efficiently cope with such incidental events. Moreover, it would be useful to include affect measure of the PANAS X both at the beginning and at the end of the study to check for possible influences of the BART performance on the affective experience of study participants in future research or at least to include it at the very beginning of the study to avoid the influence of BART performance on affect.

Lindenberger and Pötter (1998) underlined the limitations of regression mediating models in providing a reliable account of variance explained because of existing inter-correlations between predictor variables. The primary problem is that correlations between two variables that are each correlated with age may result in a spurious relationship between the two variables that does not reflect a true relationship. However, there are some counter arguments against the possibility of such spurious relationships in our analyses, showing that the effects of age on BART measures are mediated by mental speed (operationalized by the DSST). First, the zero-order correlations between mental speed and BART (see **Table 4**) and relationships between mental speed and BART in the mediation models (see **Figure 3**) preserved their signs, values, and significance levels. As demonstrated by Lindenberger and Pötter (1998), in case of spurious correlations, zero-order correlations between mediator and dependent variable may show completely different patterns than appropriate relationships within mediation models. Second, the zero-order correlations between mental speed and BART measures were calculated separately for each age group. For the older group, these correlations were substantial and significant (at least *p* < 0.05) with the average number of pumps (*r* = 0.35), the number of points (*r* = 0.29), and the number of explosions (*r* = 0.30). However, these correlations were not significant among the younger group. These patterns of correlations are consistent with theoretical arguments that larger problems of older adults (compared to younger adults) in BART performance are associated with lowered mental speed. Generally, mediation analyses using extreme age groups should be treated with caution. However, Lindenberger and Pötter (1998) advised there is nothing fundamentally wrong with using mediational analyses to show direct and indirect effects of age groups on psychological processes if there are sound theoretical arguments in favor of such a path or structural models.

In future studies, it would be important to examine other cognitive components (i.e., not only processing speed, but also working memory spans; Engle et al., 1999) and other motivational components as potential mediators of the relationship between aging and risky decision-making. Examples are the Personal Need for Structure and Need for Cognition (see Neuberg and Newsom, 1993; Cacioppo et al., 1996) and cardiovascular measures of motivation (see Hess and Ennis, 2014).

It is important, in further studies, to collect reaction times. The absence or presence of any age-related differences or interactions may be confounded by different strategies. For example, if older adults take much more time than younger adults to complete the tasks, this would lead to different interpretation than if older adults carried out the tasks very quickly.

The obvious limitations of this research are the cross-sectional character of its design and lack of comparable male participants. In some earlier experiments on risk preferences, researchers proved that in accordance with common stereotypes men indeed do prefer more risky strategies (including the determination of neurological underpinnings of these preferences, e.g., Bolla et al., 2004); while in other studies (including those which use neuroimaging techniques), no such differences were found (Galvan et al., 2007). d’Acremont and Van der Linden (2006) showed that differences in risk preferences can be explained by the choice of a specific experimental task, which seems to confirm the thesis that these differences result more from the context than biological bases (Cauffman et al., 2010). In earlier research using the BART procedure, greater risk aversion was found among women (Lejuez et al., 2002); however, this did not have a direct impact on the effectiveness of decisions that were made. Moreover, neuronal differences in this respect were also reported (Cazzell et al., 2012), which may be the result of different rates of development of brain structures in the sexes. For these reasons, we chose to focus exclusively on women. On one hand, this could be considered a limitation, but on the other hand, it eliminates possible distortions in the results by unspecified gender differences.

## Conclusion

In this study we applied the dual-process perspective of age effects on decision making. According to this theoretical perspective the influence of aging on decision making is moderated by the type of processing demands (deliberative vs. experiential). In line with predictions older participants were found to exhibit significantly higher risk aversion and lower BART performance (demanding deliberative processes) compared to their younger counterparts, adding compelling research evidence to the scarce number of studies of age effects on BART. What is important and new in the domain of aging and risky decision making, is that the effect of age on some BART measures was mediated not only by cognitive (processing speed) but also motivational (need for cognitive closure) mechanisms. We also discovered, using original experimental manipulation, that older and younger adults show similar pattern of adaptations to the initial failure (vs. success), which is most probably due to preserved efficiency of experiential processes in older age.

The aging population faces new challenges that often require making important risky decisions in unfamiliar circumstances. This study provides new knowledge about mechanisms that allow older adults to build, in a different (not necessarily worse) way, their own strategy for functioning in risk and uncertainty conditions. It turns out that in some cases, for instance when experiencing incidental failure, older adults perform as well as younger adults, which calls into question many existing stereotypes. A better understanding of the mutual roles of cognitive, motivational, and affective mechanisms (see: Peters et al., 2011; Verhaeghen et al., 2012) may lead to greater awareness among older adults of their advantages as well as disadvantages and to formation of new training programs preventing imperfect decision-making in late adulthood.

## Author Contributions

MK: design of the research, data acquisition, basic data analysis and interpretation, article drafting, final approval of the version to be published, agreement to be accountable for all aspects of the work; KR: design of the research, data acquisition, article drafting, final approval of the version to be published, agreement to be accountable for all aspects of the work; GS: supervision of the project, design of the research, advanced data analysis and interpretation, revising the work, final approval of the version to be published, agreement to be accountable for all aspects of the work.

## Conflict of Interest Statement

The authors declare that the research was conducted in the absence of any commercial or financial relationships that could be construed as a potential conflict of interest.

## References

[B1] Aklin W. M. Lejuez C. W. Zvolensky M. J. Kahler C. W. Gwadz M. (2005). Evaluation of behavioral measures of risk taking propensity with inner city adolescents. *Behav. Res. Ther* 43 215–228. 10.1016/j.brat.2003.12.007 15629751

[B2] Baltes P. B. Baltes M. M. (1990). “Psychological perspectives on successful aging: the model of selective optimization with compensation,” in *Successful Aging: Perspectives from the Behavioral Sciences*, eds Baltes P. B. Baltes M. M. (New York, NY: Cambridge University Press), 1–34.

[B3] Bargh J. A. (1994). “The Four Horsemen of automaticity: awareness, efficiency, intention, and control in social cognition,” in *Handbook of Social Cognition*, 2nd Edn, eds Wyer R. S. Jr. Srull T. K. (Hillsdale, NJ: Erlbaum), 1–40.

[B4] Baron R. M. Kenny D. A. (1986). The moderator–mediator variable distinction in social psychological research: conceptual, strategic, and statistical considerations. *J. Pers. Soc. Psychol.* 51 1173–1182. 10.1037/0022-3514.51.6.1173 3806354

[B5] Bechara A. Damasio A. R. Damasio H. Anderson S. W. (1994). Insensitivity to future consequences following damage to human prefrontal cortex. *Cognition* 50 7–15. 10.1016/0010-0277(94)90018-3 8039375

[B6] Blanchard-Fields F. Hertzog C. Stein R. Pak R. (2001). Beyond a stereotyped view of older adults’ traditional family values. *Psychol. Aging* 16 483–496. 10.1037/0882-7974.16.3.483 11554525

[B7] Bolla K. I. Eldreth D. A. Matochik J. A. Cadet J. L. (2004). Sex-related differences in a gambling task and its neurological correlates. *Cereb. Cortex* 14 1226–1232. 10.1093/cercor/bhh083 15142963

[B8] Bornovalova M. A. Cashman-Rolls A. O’Donnell J. M. Ettinger K. Richards J. B. DeWit H. (2009). Risk taking differences on a behavioral task as a function of potential reward/loss magnitude and individual differences in impulsivity and sensation seeking. *Pharmacol. Biochem. Behav.* 93 258–262. 10.1016/j.pbb.2008.10.023 19041886

[B9] Brown K. (2002). *Impact of Stock Market Decline on 50 –70 Year Old Investors.* Washington, DC: AARP, Knowledge Management.

[B10] Bruine de Bruin W. Strough J. Parker A. M. (2014). Getting older isn’t all that bad: better decisions and coping when facing sunk costs. *Psychol. Aging* 29 642–647. 10.1037/a0036308 25244483 PMC4362707

[B11] Cacioppo J. T. Petty R. E. Feinstein J. A. Jarvis W. B. G. (1996). Dispositional differences in cognitive motivation: the life and times of individuals varying in need for cognition. *Psychol. Bull.* 119 197–253. 10.1037/0033-2909.119.2.197

[B12] Carstensen L. L. (2006). The influence of a sense of time on human development. *Science* 312 1913–1915. 10.1126/science.1127488 16809530 PMC2790864

[B13] Carstensen L. L. Isaacowitz D. M. Charles S. T. (1999). Taking time seriously: a theory of socioemotional selectivity. *Am. Psychol.* 54 165–181. 10.1037/0003-066X.54.3.165 10199217

[B14] Cauffman E. Shulman E. P. Steinberg L. Claus E. Banich M. T. Graham S. (2010). Age differences in affective decision making as indexed by performance on the Iowa Gambling Task. *Dev. Psychol.* 46 193–207. 10.1037/a0016128 20053017

[B15] Cavanagh J. F. Neville D. Cohen M. X. Van de Vijver I. Harsay H. Watson P. (2012). Individual differences in risky decision-making among seniors reflect increased reward sensitivity. *Front. Neurosci.* 6:111 10.3389/fnins.2012.00111 PMC3398316 22822391

[B16] Cazzell M. Li L. Lin Z. J. Patel S. J. Liu H. (2012). Comparison of neural correlates of risk decision making between genders: an exploratory fNIRS study of the Balloon Analogue Risk Task (BART). *Neuroimage* 62 1896–1911. 10.1016/j.neuroimage.2012.05.030 22634214

[B17] Charles S. T. Carstensen L. L. (2010). Social and emotional aging. *Annu. Rev. Psychol.* 61 383–409. 10.1146/annurev.psych.093008.100448 19575618 PMC3950961

[B18] Chaubey N. P. (1974). Effect of age on expectancy of success and on risk-taking behavior. *J. Pers. Soc. Psychol.* 29 774–778. 10.1037/h0036178

[B19] Chou K.-L. Lee T. M. C. Ho A. H. Y. (2007). Does mood state change risk taking tendency in older adults? *Psychol. Aging* 22 310–318. 10.1037/0882-7974.22.2.310 17563186

[B20] Cornelis I. Van Hiel A. Roets A. Kossowska M. (2009). Age differences in conservatism: evidence on the mediating effects of personality and cognitive style. *J. Pers.* 77 51–88. 10.1111/j.1467-6494.2008.00538.x 19076995

[B21] Czarnek G. Kossowska M. Sedek G. (2015). The influence of aging on outgroup stereotypes: the mediating role of cognitive and motivational facets of deficient flexibility. *Exp. Aging Res.* 41 303–324. 10.1080/0361073X.2015.1021647 25978448

[B22] d’Acremont M. Van der Linden M. (2006). Gender differences in two decision-making tasks in a community sample of adolescents. *Int. J. Behav. Dev.* 30 352–358. 10.1177/0165025406066740

[B23] Deakin J. Aitken M. Robbins T. Sahakian B. J. (2004). Risk taking during decision-making in normal volunteers changes with age. *J. Int. Neuropsychol. Soc.* 10 590–598. 10.1017/S1355617704104104 15327737

[B24] Denburg N. L. Weller J. A. Yamada T. H. Shivapour D. M. Kaup A. R. LaLoggia A. (2009). Poor decision making among older adults is related to elevated levels of neuroticism. *Ann. Behav. Med.* 37 164–172. 10.1007/s12160-009-9094-7 19350336 PMC4028129

[B25] Dror I. E. Katona M. Mungur K. (1998). Age differences in decision making: to take a risk or not? *Gerontology* 44 67–71. 10.1159/000021986 9523216

[B26] Engle R. W. Sedek G. von Hecker U. McIntosh D. N. (eds). (2005). *Cognitive Limitations in Aging and Psychopathology.* New York, NY: Cambridge University Press.

[B27] Engle R. W. Tuholski S. W. Laughlin J. E. Conway A. R. A. (1999). Working memory, short-term memory, and general fluid intelligence: a latent-variable approach. *J. Exp. Psychol. Gen.* 128 309–331. 10.1037/0096-3445.128.3.309 10513398

[B28] Epstein S. (1994). Integration of the cognitive and the psychodynamic unconscious. *Am. Psychol.* 49 709–724. 10.1037/0003-066X.49.8.709 8092614

[B29] Fajkowska M. Marszał-Wiśniewska M. (2009). Właściwości psychometryczne Skali Pozytywnego i Negatywnego Afektu – Wersja Rozszerzona (PANAS-X). Wstkepne wyniki badań w polskiej próbie. [Psychometric properties of the Positive and Negative Affect Schedule-Expanded Form (PANAS-X). The study on a Polish sample]. *Prz. Psychol.* 52 355–388.

[B30] Finucane M. L. Mertz C. K. Slovic P. Schmidt E. S. (2005). Task complexity and older adults’ decision-making competence. *Psychol. Aging* 20 71–84. 10.1037/0882-7974.20.1.71 15769215

[B31] Frederick S. (2005). Cognitive reflection and decision making. *J. Econ. Perspect.* 19 25–42. 10.1257/089533005775196732

[B32] Frey R. Mata R. Hertwig R. (2015). The role of cognitive abilities in decisions from experience: age differences emerge as a function of choice set size. *Cognition* 142 60–80. 10.1016/j.cognition.2015.05.004 26022497

[B33] Galvan A. Hare T. Voss H. Glover G. Casey B. J. (2007). Risk-taking and the adolescent brain: who is at risk? *Dev. Sci.* 10 F8–F14. 10.1111/j.1467-7687.2006.00579.x 17286837

[B34] Hayes A. F. (2013). *Introduction to Mediation, Moderation, and Conditional Process Analysis: A Regression-Based Approach.* New York, NY: Guilford Press.

[B35] Hayes A. F. Scharkow M. (2013). The relative trustworthiness of inferential tests of the indirect effect in statistical mediation analysis: does method really matter? *Psychol. Sci.* 24 1918–1927. 10.1177/0956797613480187 23955356

[B36] Henninger D. E. Madden D. J. Huettel S. A. (2010). Processing speed and memory mediate age-related differences in decision making. *Psychol. Aging* 25 262–270. 10.1037/a0019096 20545412 PMC2896211

[B37] Hess T. M. (2000). “Aging-related constraints and adaptiation in social information processing,” in *Generative Mental Processes and Cognitive Resources: Integrative Research on Adaptation and Control*, eds Von Hecker U. Dutke S. Sedek G. (Dordrecht: Kluwer Academic Publishers), 129–156.

[B38] Hess T. M. (2014). Selective engagement of cognitive resources: motivational influences on older adults’ cognitive functioning. *Perspect. Psychol. Sci.* 9 388–407. 10.1177/1745691614527465 26173272 PMC5911399

[B39] Hess T. M. (2015). “A prospect theory-based evaluation of dual-process influences on aging and decision making: support for a contextual perspective,” in *Aging and Decision Making: Empirical and Applied Perspectives*, eds Hess T. M. Strough J. Loeckenhoff C. E. (New York, NY: Elsevier), 189–212.

[B40] Hess T. M. Ennis G. E. (2014). Assessment of adult age differences in task engagement: the utility of systolic blood pressure. *Motiv. Emot.* 38 844–854. 10.1007/s11031-014-9433-2 25530642 PMC4270001

[B41] Hess T. M. Queen T. L. Ennis G. E. (2013). Age and self-relevance effects on information search during decision making. *J. Gerontol. B Psychol.* 68 703–711. 10.1093/geronb/gbs108 PMC3859358 23197342

[B42] Hess T. M. Queen T. L. Patterson T. (2012). To deliberate or not to deliberate: interactions between age, task characteristics, and cognitive activity on decision making. *J. Behav. Decis. Mak.* 25 29–40. 10.1002/bdm.711 24532954 PMC3923383

[B43] Hopko D. R. Lejuez C. W. Daughters S. B. Aklin W. M. Osborne A. Simmons B. L. (2006). Construct validity of the Balloon Analogue Risk Task (BART): relationship with MDMA use by inner-city drug users in residential treatment. *J. Psychopathol. Behav. Assess.* 28 95–101. 10.1007/s10862-006-7487-5

[B44] Hunt M. K. Hopko D. R. Bare R. Lejuez C. W. Robinson E. V. (2005). Construct validity of the Balloon Analog Risk Task (BART): associations with psychopathy and impulsivity. *Assessment* 12 416–428. 10.1177/1073191105278740 16244122

[B45] Kahneman D. (2003). A perspective on judgment and choice: mapping bounded rationality. *Am. Psychol.* 58 697–720. 10.1037/0003-066X.58.9.697 14584987

[B46] Kossowska M. (2007). Motivation toward closure and cognitive processes: an individual differences approach. *Pers. Indiv. Diff.* 43 2149–2158. 10.1016/j.paid.2007.06.027

[B47] Kossowska M. Hanusz K. Trejtowicz M. (2012a). Skrócona wersja Skali Potrzeby Poznawczego Domknikecia. Dobór pozycji i walidacja skali. [Short version of the Need for Cognitive Closure Scale: items selection and scale validation]. *Psychol. Społ.* 7 89–99.

[B48] Kossowska M. Jaśko K. Bar-Tal Y. Szastok M. (2012b). The relationship between need for closure and memory for schema-related information among younger and older adults. *Neuropsychol. Dev. Cogn. B Aging Neuropsychol. Cogn.* 19 283–300. 10.1080/13825585.2011.632617 22176023

[B49] Kossowska M. Orehek E. Kruglanski A. W. (2010). “Motivation towards closure and cognitive resources: an individual differences approach,” in *Handbook of Individual Differences in Cognition: Attention, Memory and Executive Control*, eds Gruszka A. Mathews G. Szymura B. (New York, NY: Springer Science + Business Media), 369–382.

[B50] Kovalchik S. Camerer C. F. Grether D. M. Plott C. R. Allman J. M. (2005). Aging and decision making: a comparison between neurologically healthy elderly and young individuals. *J. Econ. Behav. Organ.* 58 79–94. 10.1016/j.jebo.2003.12.001

[B51] Kruglanski A. Webster D. (1996). Motivated closing of the mind: “Seizing” and “freezing”. *Psychol. Rev.* 2 263–283. 10.1037/0033-295X.103.2.263 8637961

[B52] Kruglanski A. W. (2004). *The Psychology of Closed Mindedness.* New York, NY: Psychology Press.

[B53] Lauriola M. Panno A. Levin I. P. Lejuez C. W. (2014). Individual differences in risky decision making: a meta-analysis of sensation seeking and impulsivity with the Balloon Analogue Risk Task. *J. Behav. Decis. Mak.* 27 20–36. 10.1002/bdm.1784

[B54] Lejuez C. W. Aklin W. M. Jones H. A. Richards J. B. Strong D. R. Kahler C. W. (2003). The Balloon Analogue Risk Task (BART) differentiates smokers and nonsmokers. *Exp. Clin. Psychopharmacol.* 11 26–33. 10.1037/1064-1297.11.1.26 12622341

[B55] Lejuez C. W. Read J. P. Kahler C. W. Richards J. B. Ramsey S. E. Stuart G. L. (2002). Evaluation of a behavioral measure of risk taking: the Balloon Analogue Risk Task (BART). *J. Exp. Psychol. Appl.* 8 75–84. 10.1037/1076-898X.8.2.75 12075692

[B56] Lindenberger U. Pötter U. (1998). The complex nature of unique and shared effects in hierarchical linear regression: implications for developmental psychology. *Psychol. Methods* 3 218–230. 10.1037/1082-989X.3.2.218

[B57] MacPherson S. E. Phillips L. H. Della Sala S. (2002). Age, executive function and social decision making: a dorsolateral prefrontal theory of cognitive aging. *Psychol. Aging* 17 598–609. 10.1037/0882-7974.17.4.598 12507357

[B58] Mata R. Josef A. K. Samanez-Larkin G. R. Hertwig R. (2011). Age differences in risky choice: a meta-analysis. *Ann. N. Y. Acad. Sci.* 1235 18–29. 10.1111/j.1749-6632.2011.06200.x 22023565 PMC3332530

[B59] Mata R. Schooler L. J. Rieskamp J. (2007). The aging decision maker: cognitive aging and the adaptive selection of decision strategies. *Psychol. Aging* 22 796–810. 10.1037/0882-7974.22.4.796 18179298

[B60] Neuberg S. L. Newsom J. (1993). Individual differences in chronic motivation to simplify: personal need for structure and social-cognitive processing. *J. Pers. Soc. Psychol.* 65 113–131. 10.1037/0022-3514.65.1.113

[B61] Otto A. R. Fleming S. M. Glimcher P. W. (2016). Unexpected but incidental positive outcomes predict real-world gambling. *Psychol. Sci.* 27 299–311. 10.1177/0956797615618366 26796614

[B62] Peters E. Dieckmann N. E. Weller J. (2011). “Age differences in complex decision making,” in *Handbook of the Psychology of Aging*, 7th Edn, eds Schaie K. W. Willis S. L. Schaie K. W. Willis S. L. (San Diego, CA: Elsevier), 133–151.

[B63] Peters E. Hess T. M. Västfjäll D. Auman C. (2007). Adult age differences in dual information processes: implications for the role of affective and deliberative processes in older adults’ decision making. *Perspect. Psychol. Sci.* 2 1–23. 10.1111/j.1745-6916.2007.00025.x 26151915

[B64] Queen T. L. Hess T. M. (2010). Age differences in the effects of conscious and unconscious thought in decision making. *Psychol. Aging* 25 251–261. 10.1037/a0018856 20545411 PMC2896215

[B65] Queen T. L. Hess T. M. Ennis G. E. Dowd K. Grühn D. (2012). Information search and decision making: effects of age and complexity on strategy use. *Psychol. Aging* 27 817–824. 10.1037/a0028744 22663157 PMC3435436

[B66] Roets A. Kruglanski A. W. Kossowska M. Pierro A. Hong Y.-Y. (2015). “The motivated gatekeeper of our minds,” in *Advances in Experimental Social Psychology* Vol. 52 eds Olson J. M. Zanna M. P. (London: Elsevier), 221–283.

[B67] Rolison J. J. Hanoch Y. Wood S. (2012). Risky decision making in younger and older adults: the role of learning. *Psychol. Aging* 27 129–140. 10.1037/a0024689 21767022

[B68] Salthouse T. (2006). Mental exercise and mental aging. *Perspect. Psychol. Sci.* 1 68–87. 10.1111/j.1745-6916.2006.00005.x 26151186

[B69] Salthouse T. (2012). Consequences of age-related cognitive decline. *Ann. Rev. Psychol.* 63 201–226. 10.1146/annurev-psych-120710-100328 21740223 PMC3632788

[B70] Salthouse T. A. (1996). The processing-speed theory of adult age differences in cognition. *Psychol. Rev.* 103 403–428. 10.1037/0033-295X.103.3.403 8759042

[B71] Salthouse T. A. (2001). Structural models of the relations between age and measures of cognitive functioning. *Intelligence* 29 93–115. 10.1016/j.cortex.2010.01.001

[B72] Samanez-Larkin G. R. Wagner A. D. Knutson B. (2011). Expected value information improves financial risk taking across the adult life span. *Soc. Cogn. Affect. Neurosci.* 6 207–217. 10.1093/scan/nsq043 20501485 PMC3073388

[B73] Seaman K. L. Stillman C. M. Howard D. V. Howard J. H. Jr. (2015). Risky decision-making is associated with residential choice in healthy older adults. *Front. Psychol.* 6:1192 10.3389/fpsyg.2015.01192 PMC4531216 26322000

[B74] Sedek G. Kossowska M. Rydzewska K. (2014). The importance of adult life-span perspective in explaining variations in political ideology. *Behav. Brain Sci.* 37 329–330. 10.1017/S0140525X13002732 24970451

[B75] Sedek G. Verhaeghen P. Martin M. (eds) (2013). *Social and Motivational Compensatory Mechanisms of Age-related Cognitive Decline. Special Issue of Aging, Neuropsychology, and Cognition.* Hove: Psychology Press.

[B76] Sedek G. von Hecker U. (2004). Effects of subclinical depression and aging on generative reasoning about linear orders: same or different processing limitations? *J. Exp. Psychol. Gen.* 133 237–260. 10.1037/0096-3445.133.2.237 15149252

[B77] Statistic Brain Research Institute (2015). *CEO Statistics.* Available at: http://www.statisticbrain.com/ceo-statistics/

[B78] Thornton W. J. L. Dumke H. (2005). Age differences in everyday problem solving and decision making effectiveness: a meta-analytic review. *Psychol. Aging* 20 85–99. 10.1037/0882-7974.20.1.85 15769216

[B79] Verhaeghen P. Martin M. Sedek G. (2012). Reconnecting cognition in the lab and cognition in real life: the role of compensatory social and motivational factors in explaining how cognition ages in the wild. *Neuropsychol. Dev. Cogn. B Aging Neuropsychol. Cogn.* 19 1–12. 10.1080/13825585.2011.645009 22313173 PMC3775600

[B80] von Helversen B. Mata R. (2012). Losing a dime with a satisfied mind: positive affect predicts less search in sequential decision making. *Psychol. Aging* 4 825–839. 10.1037/a0027845 22449028

[B81] Watson D. Clark L. A. (1994). *The PANAS-X: Manual for the Positive and Negative Affect Schedule-Expanded Form.* Available at: http://ir.uiowa.edu/

[B82] Wechsler D. (1981). *WAIS-R Manual: Wechsler Adult Intelligence Scale-Revised.* New York, NY: The Psychological Corporation.

[B83] Wood S. Busemeyer J. Koling A. Cox C. R. Davis H. (2005). Older adults as adaptive decision makers: evidence from the Iowa Gambling Task. *Psychol. Aging* 20 220–225. 10.1037/0882-7974.20.2.220 16029086

